# Desert crossing strategies of migrant songbirds vary between and within species

**DOI:** 10.1038/s41598-019-56677-4

**Published:** 2019-12-27

**Authors:** Frédéric Jiguet, Malcolm Burgess, Kasper Thorup, Greg Conway, José Luis Arroyo Matos, Lee Barber, John Black, Niall Burton, Joan Castelló, Gary Clewley, José Luis Copete, Michel Alexandre Czajkowski, Svein Dale, Tony Davis, Valery Dombrovski, Mike Drew, Jaanus Elts, Vicky Gilson, Emilienne Grzegorczyk, Ian Henderson, Michael Holdsworth, Rob Husbands, Romain Lorrilliere, Riho Marja, Simonas Minkevicius, Caroline Moussy, Peter Olsson, Alejandro Onrubia, Marc Pérez, Joseph Piacentini, Markus Piha, Jean-Marc Pons, Petr Procházka, Marko Raković, Harriet Robins, Tuomas Seimola, Gunnar Selstam, Michał Skierczyński, Jan Sondell, Jean-Claude Thibault, Anders P. Tøttrup, Justin Walker, Chris Hewson

**Affiliations:** 1 CESCO, UMR7204 MNHN-CNRS-Sorbonne Université, CP135, 43 Rue Buffon, 75005 Paris, France; 2 RSPB Centre for Conservation Science, The Lodge, Sandy, SG19 2DL United Kingdom; 3 Center for Macroecology, Evolution and Climate, Natural History Museum of Denmark, University of Copenhagen, Universitetsparken 15, 2100 Copenhagen, Denmark; 4 British Trust for Ornithology, The Nunnery, Thetford, Norfolk, IP24 2PU United Kingdom; 5 Grupo Ibérico de Anillamiento (GIA), C/Daoiz y Velarde, 49, Bajo, 24006 León Spain; 6 Handbook of the Birds of the World Alive, Lynx Edicions, Montseny 8, 08193 Bellaterra, Spain; 7 OMPO, 59 rue Ampère, 75017 Paris, France; 8 Faculty of Environmental Sciences and Natural Resource Management, Norwegian University of Life Sciences, P.O. Box 5003, NO–1432 Ås, Norway; 9 Institute of Zoology, National Academy of Sciences, Academichnaya 27, 220072 Minsk, Belarus; 10 Biodiversity & Catchment, Anglian Water Services Limited, Lancaster House, Lancaster Way, Ermine Business Park, Huntingdon, Cambridgeshire PE29 6XU United Kingdom; 11 Estonian Ornithological Society, Veski 4, 51005 Tartu, Estonia & Department of Zoology, Institute of Ecology and Earth Sciences, University of Tartu, 46 Vanemuise St., 51014 Tartu, Estonia; 12 3 Oakhill Road, Mitcheldean, Gloucestershire, GL17 0BN United Kingdom; 13 MTA Centre for Ecological Research, Institute of ecology and Botany, “Lendület” Landscape and Conservation Ecology, Alkotmány u. 2-4, 2163 Vácrátót, Hungary; 14 Pajautos st. 11-40, LT, 06203 Vilnius, Lithuania; 15 Centre for Environmental and Climate Research (CEC), Ekologihuset, Sölvegatan 37, Lund, Sweden; 16 Migres Foundation, International Bird Migration Center (CIMA), N-340, Km 85, P.O. Box 152, 11380 Tarifa, Cádiz Spain; 17 Nostra Senyora de Montserrat 19, 08756 La Palma de Cervelló, Spain; 18 Piedalbuccio, 20232 Oletta, France; 19 Finnish Museum of Natural History LUOMUS, P.O. Box 17 (Pohjoinen Rautatiekatu 13), FI-00014 University of Helsinki, Helsinki, Finland; 20 Institut Systématique, Evolution, Biodiversité (ISYEB, UMR7205), MNHN-CNRS-SU-EPHE, 57 Rue Cuvier, CP50, 75005 Paris, France; 21 The Czech Academy of Sciences, Institute of Vertebrate Biology, Květná 8, CZ-603 65, Brno, Czech Republic; 22 Natural History Museum of Belgrade, Njegoševa, 51 Serbia; 23 New buildings, Howle Hill, Ross on Wye, Herefordshire, HR9 5RD United Kingdom; 24 Natural Resources Institute Finland (Luke), Natural Resources, Latokartanonkaari 9, 00790 Helsinki, Finland; 25 Department of Agricultural Research in Northern Sweden, Swedish University of Agricultural Sciences and Department of Molecular Biology, University of Umeå, 901 85 Umeå, Sweden; 26 Department of Behavioural Ecology, Adam Mickiewicz University, Poznan, Poland, Dziczenie.pl, Gruszki Poland; 27 Kvismare Bird Observatory, Rulleuddsvägen 10, S-178 51 Ekerö, Sweden; 28 Natural History Museum of Denmark, University of Copenhagen, Universitetsparken 15, 2100 Copenhagen, Denmark

**Keywords:** Animal migration, Behavioural ecology

## Abstract

Each year, billions of songbirds cross large ecological barriers during their migration. Understanding how they perform this incredible task is crucial to predict how global change may threaten the safety of such journeys. Earlier studies based on radar suggested that most songbirds cross deserts in intermittent flights at high altitude, stopping in the desert during the day, while recent tracking with light loggers suggested diurnal prolongation of nocturnal flights and common non-stop flights for some species. We analyzed light intensity and temperature data obtained from geolocation loggers deployed on 130 individuals of ten migratory songbird species, and show that a large variety of strategies for crossing deserts exists between, but also sometimes within species. Diurnal stopover in the desert is a common strategy in autumn, while most species prolonged some nocturnal flights into the day. Non-stop flights over the desert occurred more frequently in spring than in autumn, and more frequently in foliage gleaners. Temperature recordings suggest that songbirds crossed deserts with flight bouts performed at various altitudes according to species and season, along a gradient ranging from low above ground in autumn to probably >2000 m above ground level, and possibly at higher altitude in spring. High-altitude flights are therefore not the general rule for crossing deserts in migrant songbirds. We conclude that a diversity of migration strategies exists for desert crossing among songbirds, with variations between but also within species.

## Introduction

How small migrant songbirds cross large ecological barriers, such as deserts and seas, has been investigated by ecologists for decades^1,2^. Ornithologists initially assumed that most migrants cross large ecological barriers by undertaking non-stop flights. Field observations and radar studies later suggested that nocturnally migrating passerines exhibit intermittent rather than non-stop flights to cross the Sahara^3^. The recent development of light-level geolocation tracking^4^ challenged this proposal by revealing that some songbirds perform 40–60h non-stop flights to cross deserts^5^, as others do to cross oceans^6,7^. Prolonging nocturnal flight into daytime was detected by radar studies^8^, and appears to be common^9^, while non-stop flight has even been proposed as the prevalent strategy to cross large ecological barriers^5^. How small migrant songbirds cross such barriers is thus still questioned, such as the potential predominance of a general strategy among a variety of species, or a diversity of individual strategies.

Migrant songbirds are generally thought to perform barrier crossing flights at high altitude, in order to minimize energy costs^10^. For example, nocturnal flights are performed at >1000 m above sea level (a.s.l.) in great reed warblers *Acrocephalus arundinaceus* and Eurasian hoopoes *Upupa epops*, with most tracked warblers reaching heights up to 5000 m a.s.l.^11^. In continental Europe, Doppler weather radars locate the highest densities of active nocturnal migrant birds at around 2000 m above ground level (a.g.l.)^12^, though such radars are not able to differentiate between species. Above the Sahara, radar studies detected highest densities of passerine diurnal migration around 3500–4000m in autumn, up to 5000m in spring^10^.

Here, by analyzing anomalies in light intensity and temperature data recorded by geolocation loggers carried by 130 individuals of ten long-distance Palearctic migrant songbird species during their migration, we studied species diurnal migration patterns when crossing deserts in autumn (134 tracks) and spring (80 tracks). Based on conflicting earlier evidence and the diversity of species we studied, we expect to find a variety of strategies among these species, with no single rule for desert crossing in migratory songbirds, ranging from complete diurnal stopover to continuous non-stop flight. We further aim at broadly estimating relative flying altitudes of seven species from temperature differentials recorded during barrier crossings, and discuss the implication of migration altitude in the context of climate change.

**Figure 1:**
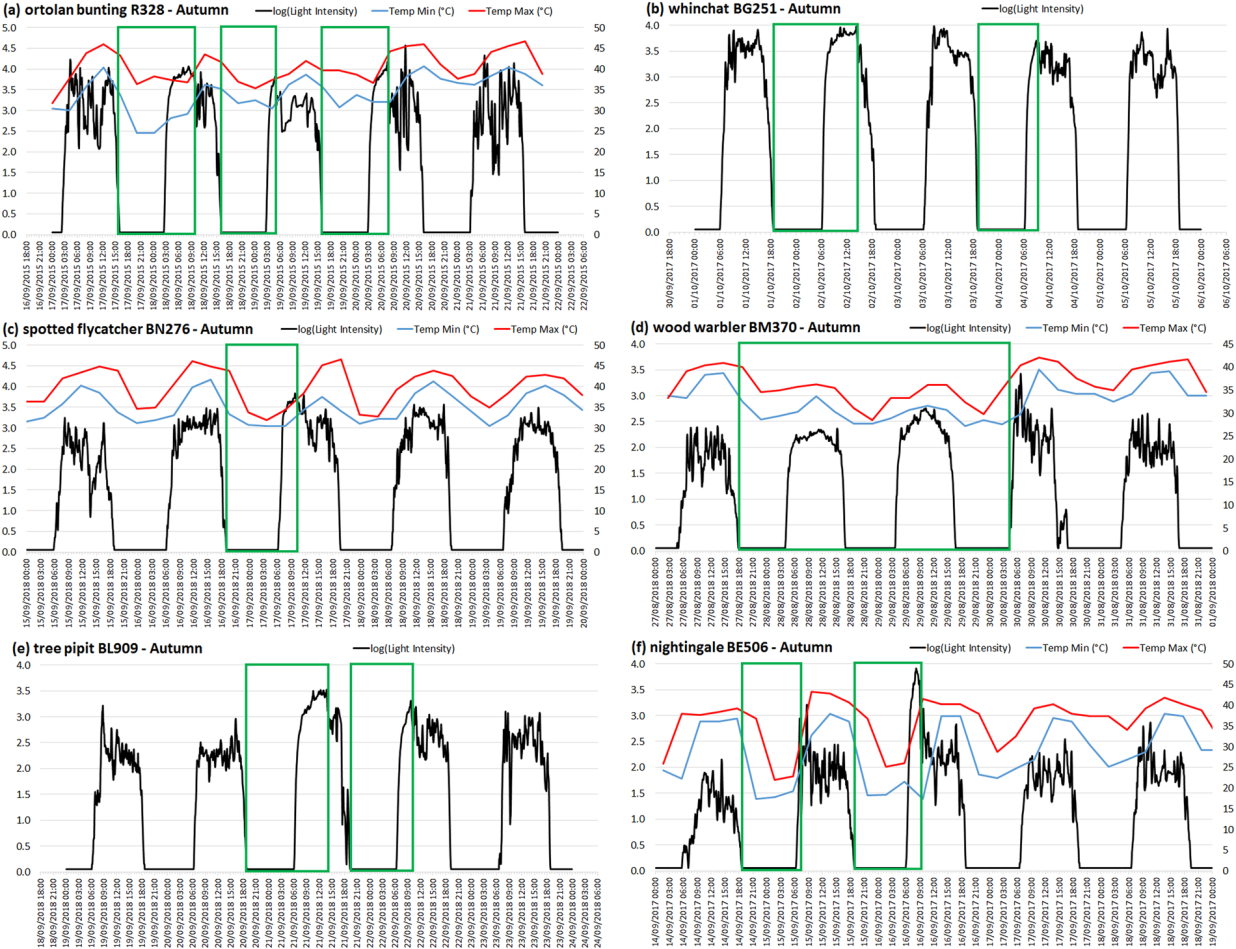
Light intensity (log-transformed), minimal and maximal temperatures (if available) recorded by geolocators during autumn desert crossing for six individuals of different species. Green rectangles represent possible flight bouts. (**a**) ortolan bunting R328: FLP during three consecutive days; (**b**) whinchat BG251: long FLP on second day and short FLP on fourth day, so not on consecutive days; (**c**) spotted flycatcher BN276: FLP in the third day, with lower temperatures in the morning of that day. (**d**) wood warbler BM370: non-stop flights during three nights and two days, revealed by complete FLPs and low temperatures; (**e**) tree pipit BL909: third and fourth days with obvious FLPs; no temperature data available. (**f**) nightingale BE506: two nights with large drops in minimal and maximal temperatures, the second followed by a short FLP, corresponding to a low minimal temperature saved at 9:39, so recorded during the four previous hours (5:39–9:39), while sunrise occurred at 6:00.

**Figure 2:**
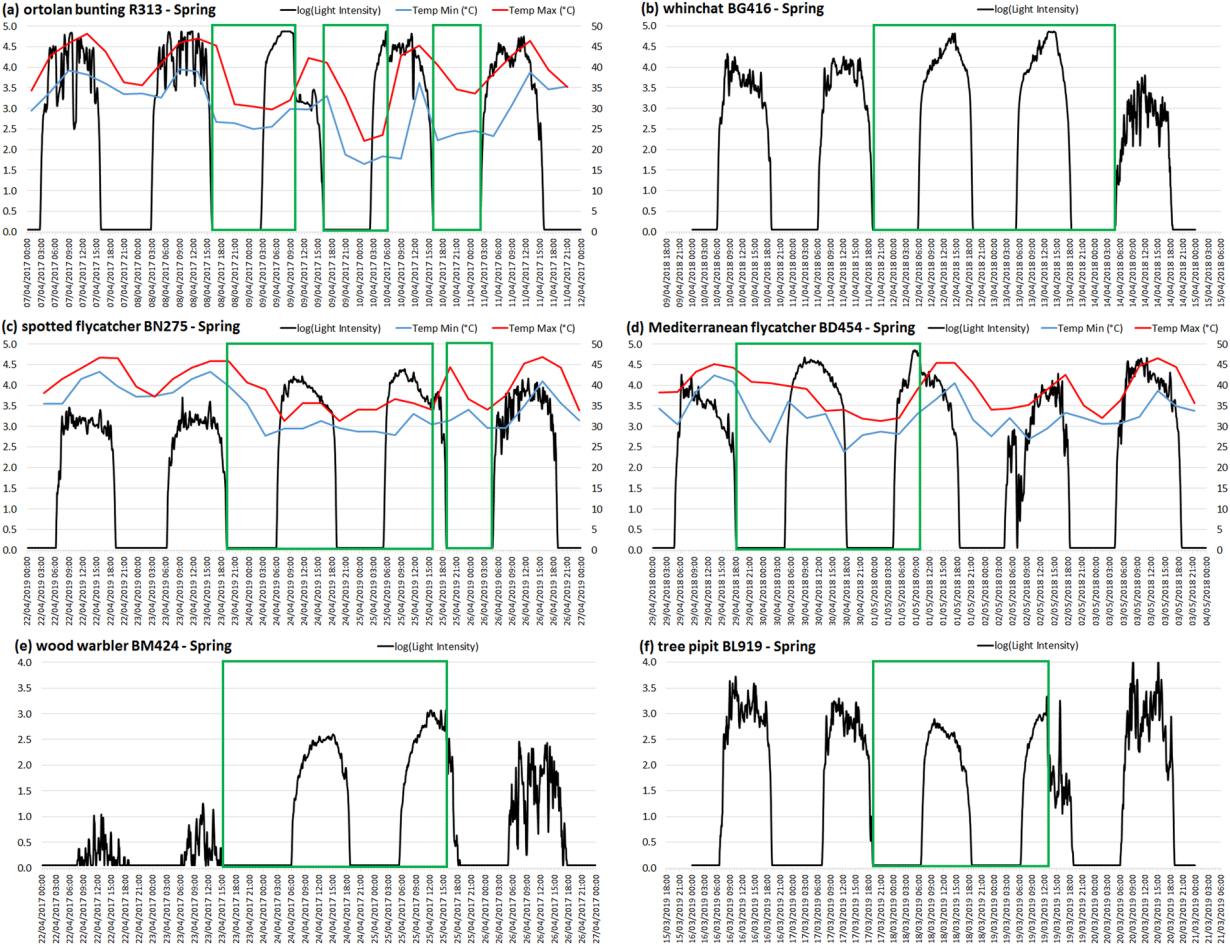
Light intensity (log-transformed) and minimum and maximum temperature (if available) recorded by geolocators during spring desert crossing, for six individuals of different species. Green rectangles represent possible flight bouts. (**a**) ortolan bunting R313: FLPs over two consecutive days, and temperature drops in the third night revealing a third night of active migration at a relatively high altitude; (**b**) whinchat BG416: two full-day FLPs during two consecutive days; species foraging in the open, like whinchat and flycatchers, where a full-day FLP is harder to distinguish from usual patterns than in species foraging in bushes and trees, like wood warbler and nightingale; (**c**) spotted flycatcher BN275: non-stop flight during two nights and a complete day, followed by a prolongation during most of the second day, for a flight bout that lasted potentially up to 45 hours, while the low temperatures during the following night reveal another nocturnal flight bout; (**d**) Mediterranean flycatcher BD454 spring: full-day FLP on the second day, and FLP in the morning of the third day, for a non-stop flight bout that lasted potentially up to 40 hours; (**e**) wood warbler BM424: a non-stop flight that lasted potentially up to 48 hours; (**f**) tree pipit BL919: a non-stop flight that lasted potentially up to 40 hours.

Looking at light intensity and temperature data recorded by the loggers, we searched for patterns showing that individual birds prolonged a nocturnal migration flight into the day. A prolonged flight is revealed by a full light pattern (FLP) at least in the morning^9^, and generally (if recorded) a lower minimal temperature recorded during the four previous hours. This is followed by an abrupt change in the light intensity pattern and an increase in temperature after the bird has landed, as the tag’s light sensor experiences greater, more variable shading compared to in flight and the temperature sensor responds to warmer ambient temperatures on the ground and a reduction in wind chill (Figs. 1 & 2). A flight prolonged over a complete day is revealed by a full-day FLP and low minimal and maximal temperatures during the whole day, including at noon (Fig. 2). A full-day stopover is revealed by an absence of FLP and large drops in nocturnal temperatures the previous and next nights during active migration, or by the absence of both FLP and temperature drops during the complete migration season when geolocation proves that the individual crossed a desert. The ten tracked migratory songbird species are: ortolan bunting *Emberiza hortulana* (n = 26 individuals), whinchat *Saxicola rubetra* (n = 20), spotted flycatcher *Muscicapa striata* (n = 22) and its sister species Mediterranean flycatcher *Muscicapa tyrrhenica* (n = 5), willow warbler *Phylloscopus trochilus* (n = 15), wood warbler *Phylloscopus sibilatrix* (n = 3), Eurasian reed warbler *Acrocephalus scirpaceus* (n = 11), tree pipit *Anthus trivialis* (n = 9), rufous-tailed scrub-robin *Cercotrichas galactotes* (n = 1) and nightingale *Luscinia megarhynchos* (n = 18).

## Results

### Non-stop desert crossing

**Figure 3:**
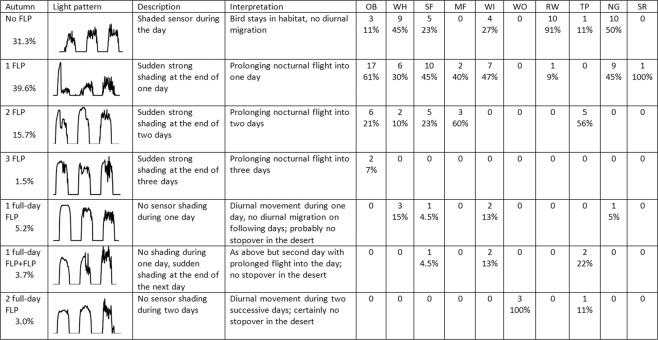
Detailed autumn overview of daytime light pattern anomalies (FLP) recorded by geolocators while birds were crossing a desert. Each category is accompanied by a representative figure of recorded light intensities, description of the anomaly and the most plausible interpretation, numbers of individuals and species-specific % of occurrence, in line with previous publications^9^. OB: ortolan bunting, WH: whinchat, SF: spotted flycatcher, MF: Mediterranean flycatcher, WI: willow warbler, WO: wood warbler, RW: Eurasian reed warbler, TP: tree pipit, NG: nightingale, SR: rufous-tailed scrub-robin. The first four categories total 88.1% of tracked individuals (stopovers in the desert), the last three categories 11.9% (probable non-stop flight over the desert.

**Figure 4:**
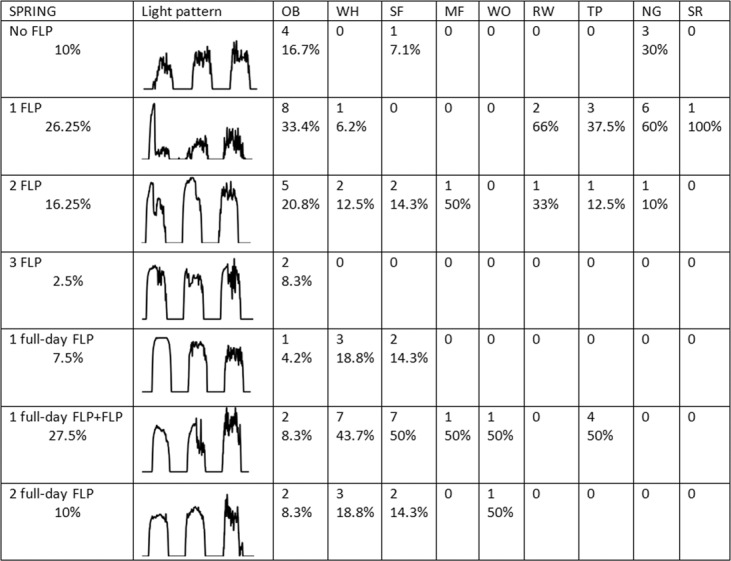
Detailed spring overview of daytime light pattern anomalies (FLP) recorded by geolocators while birds were crossing a desert. Each category is accompanied by a representative figure of recorded light intensities, numbers of individuals and species-specific % of occurrence, in line with previous publications^9^. OB: ortolan bunting, WH: whinchat, SF: spotted flycatcher, MF: Mediterranean flycatcher, WO: wood warbler, RW: Eurasian reed warbler, TP: tree pipit, NG: nightingale, SR: rufous-tailed scrub-robin. The first four categories totalize 55% of tracked individuals (stopovers in the desert), the last three categories 45% (probable non-stop flight over the desert) – see Fig. 3 for the description and interpretation of light patterns.

Most songbird individuals stopped migrating during the day while crossing a desert barrier, in both autumn and spring. In autumn, only 11% of individuals studied performed a non-stop barrier crossing flight (Fig. 3). This group comprised all wood warblers, one third of tree pipits, one quarter of willow warblers, and one out of ten whinchats and spotted flycatchers. In spring, 44% of the individuals performed a non-stop flight over at least a complete day, often followed by a diurnal prolongation of the next nocturnal flight (Fig. 2 & 4) – probably to complete the desert crossing in a single non-stop flight. This group comprised few ortolan buntings, half the tree pipits, most spotted flycatchers and whinchats, and all wood warblers.

We tested if the season and the date of barrier crossing within the season could predict the probability to perform a non-stop flight over the desert (see Methods for details). We found a strong seasonal component (non-stop flights more frequent in spring, P < 0.001) and large differences between species (P < 0.001), but no influence of the date (P = 0.74).

### Diurnal extension of nocturnal flights during intermittent desert crossings

**Figure 5:**
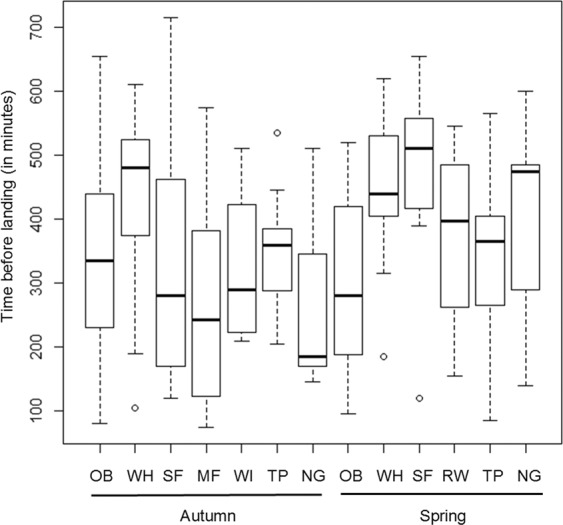
Boxplot of the time of landing after sunrise for days with FLPs not lasting a complete day, during the autumn (7 species) and spring (6 species) migrations. Average, box of 25^th^ and 75^th^ quartiles, and 5%-95% confidence intervals, with dots showing potential outliers. Labels of the x-axis are codes for species names: OB: ortolan bunting, WH: whinchat, SF: spotted flycatcher, MF: Mediterranean flycatcher, WI: willow warbler, RW: Eurasian reed warbler, TP: tree pipit, NG: nightingale.

Of the 89% and 56% of migration tracks in autumn and spring, respectively, where individuals landed whilst crossing a desert, 78% and 90% showed at least one FLP. Thus they prolonged some nocturnal migration flights into at least one morning before stopping for the rest of the day, either in the desert or at the end of the crossing (Figs. 3 and 4). In autumn, this included the majority of the buntings, flycatchers, warblers, pipits and chats (nightingales and rufous-tailed scrub-robin). In spring, this included all whinchats, warblers, pipits, and most buntings, flycatchers and chats. Flight extension into the day tended to occur only on one or two days in both autumn and spring, lasting 3 to 7 hours after sunrise (Fig. 5). We found no difference in the duration of prolonged diurnal flights (excluding full-day FLPs) in autumn for the seven species with FLPs and at least five tracked individuals (generalized linear model; Fig. 5; F_6,93_ = 1.29, P = 0.27). We did, however, find differences between species in spring for the six species with at least four tracked individuals (Fig. 5; F_5,62_ = 3.53, P = 0.007), with whinchats and spotted flycatchers revealing longer diurnal prolongations of nocturnal flights.

### Intra-specific diversity of strategies

Wood Warbler was the only species for which all individuals undertook a non-stop flight to cross the Sahara in both autumn and spring crossings, over two nights and two days in all cases. At the other extreme, all Eurasian reed warblers and all nightingales (except one in autumn) stopped in the desert in both seasons, while all Mediterranean flycatchers stopped in the desert in autumn. Willow warblers revealed the most diverse strategies in autumn, with four individuals performing only nocturnal flights (no FLP), seven individuals prolonging nocturnal flights into the morning (FLP), and four individuals performing non-stop flights (FLP over a complete day). We also observed these three main strategies for at least one individual of four other species in autumn (whinchat, spotted flycatcher, tree pipit and nightingale).

### Temperature differentials

**Figure 6:**
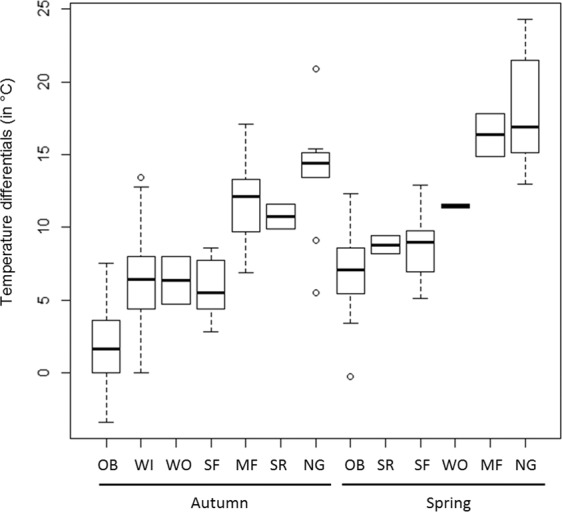
Boxplot showing the differences (average, box of 25^th^ and 75^th^ quartiles, and 5%-95% confidence intervals) in maximum temperature recorded on a day of prolonged nocturnal flight compared to the maximum temperature recorded at the same hour the previous day, or on the next day that occurred without a prolonged flight during daylight. Labels of the x-axis are codes for species names and seasons: OB: ortolan bunting, WI: willow warbler, WO: wood warbler, SF: spotted flycatcher, MF: Mediterranean flycatcher, SR: rufous-tailed scrub-robin, NG: nightingale.

For seven species, light loggers also recorded temperature data, which further confirmed the diurnal prolongation of nocturnal flights into the morning. Examining temperature differentials between migratory flights and equivalent times when stationary (Table 1; see Methods for details), we found a gradient of temperature differentials (Fig. 6), while temperature differentials were larger in spring than in autumn (generalized linear model, season effect after accounting for species identity, t_1,131_ = 7.4, P < 0.001; estimate ± s.d. = 4.1 ± 0.6 °C). For the two closely related flycatchers, we found statistically different temperature differentials between seasons (t_34_ = 2.26, P = 0.03), and larger temperature differentials in Mediterranean flycatchers (t_34_ = −5.69, P < 0.001). Temperature differentials might represent a broad proxy for flight altitude, though we discuss further below the limits to such a relationship.

**Table 1:** Temperature differentials between consecutive days with and without prolonged nocturnal flights while desert crossing, per species and season. Min and Max values reported here are the minimal and maximal estimated temperature differentials. Sample size refers to the number of day pairs.

Season	Species	Mean ± s.d. (°C)	Min-Max (°C)	N
Autumn	ortolan bunting	1.8 ± 2.9	−3.4/7.5	33
	spotted flycatcher	5.9 ± 1.8	2.8/8.6	10
	Mediterranean flycatcher	12.0 ± 3.2	6.9/17.1	10
	willow warbler	6.4 ± 3.8	0/13.4	17
	wood warbler	6.4 ± 2.3		2
	nightingale	14.1 ± 4.2	6.8/24.3	26
	rufous-tailed scrub-robin	10.8 ± 1.2		2
Spring	ortolan bunting	7.1 ± 2.6	−0.3/12.3	27
	spotted flycatcher	8.5 ± 2.1	5.1/12.9	15
	Mediterranean flycatcher	11.5 ± 0.2		2
	wood warbler	8.8 ± 0.8		2
	nightingale	17.6 ± 3.4	13.0/24.3	14
	rufous-tailed scrub-robin	16.4 ± 2.1		2

### Spatial and temporal occurrences of FLP

**Figure 7:**
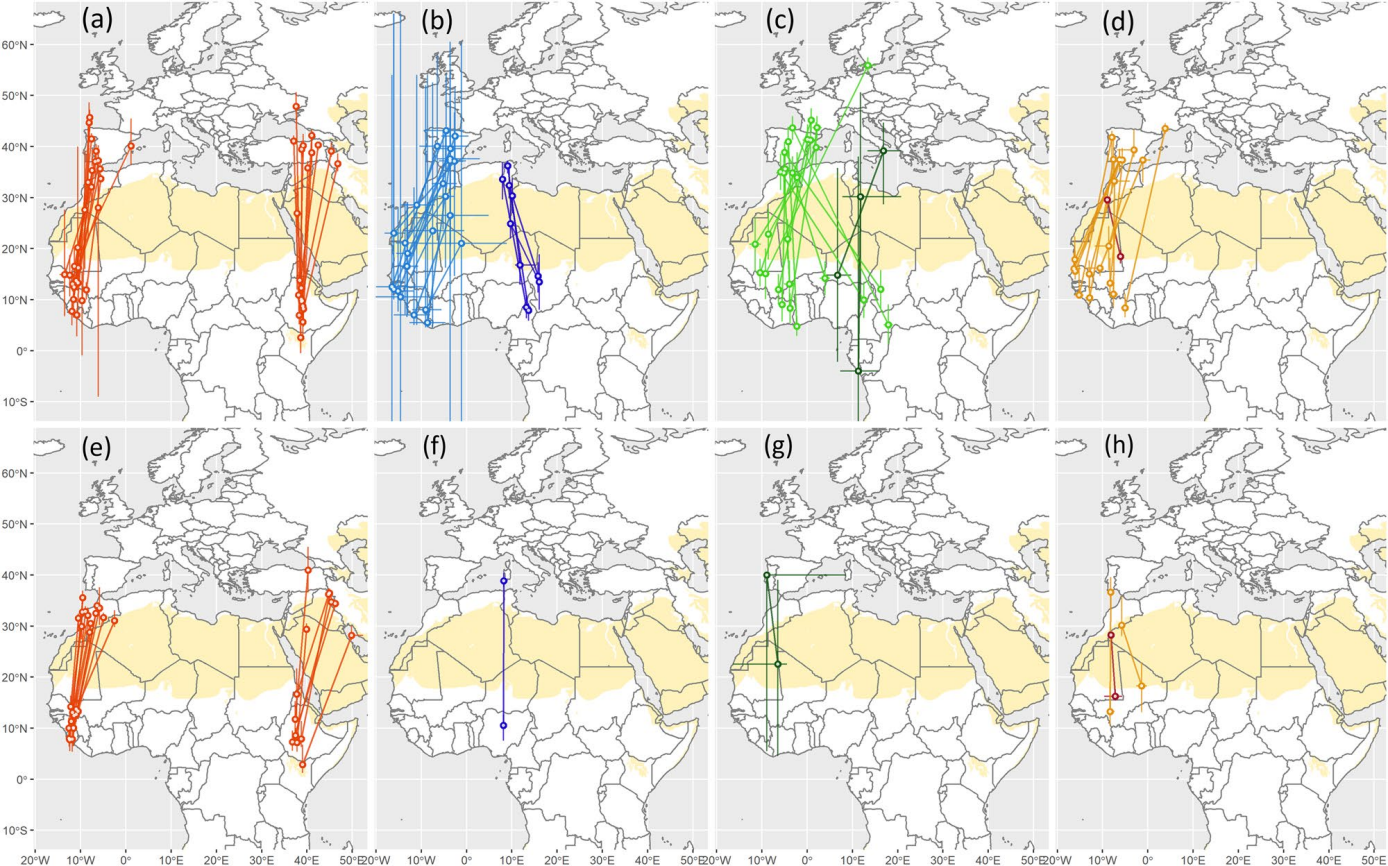
Stationary sites of individual birds (median ±25th/75th or 20th/80th percentiles of location estimates) just prior and after the occurrence of the full light pattern(s) detected during autumn (**a**–**d**) and spring (**e**–**h**) migration; red = ortolan bunting (**a**,**e**); light blue = spotted flycatcher, dark blue = Mediterranean flycatcher (b,f); light green = willow warbler, dark green = wood warbler (**c**,**g**); orange = Eurasian reed warbler, dark red = rufous-tailed scrub-robin (**d**,**h**).

For all species and individuals with coordinates estimated even during equinox periods, previous and subsequent stationary areas, as determined from light intensity data^13^, were always north and south of a desert (Fig. 7), the Sahara for individuals using the western and central flyways (ten species), or the Arabian desert for ortolan buntings using the eastern flyway^14^. Table S1 reports the first and last dates with FLP for each species and season (with individual details in Table S2).

## Discussion

By examining light level and temperature data from ten songbird species over two different desert barrier crossings, in autumn and spring migrations, we conclude that there are multiple strategies for barrier crossing. These vary between the species and between the seasonal migrations, but also between individuals within a species. However, both in autumn and spring, the most frequent strategy adopted is to stop over in the desert during the day, generally after a diurnal prolongation of the nocturnal flight for 2–10 hours (Fig. 5). The diversity of migration strategies when crossing ecological barriers probably reflect the diversity of species-specific strategies and of individual decisions. Apparent non-stop flights over the desert were nonetheless observed in seven species (all except reed warblers and chats) and were universal among wood warblers. They were more frequent in spring than in autumn. This may be an adaptation for faster spring migration and earlier breeding grounds arrival^15^. We did not, however, find a higher occurrence of non-stop crossing as the spring season advances.

One previous study^7^ suggested that non-stop flights could be a common strategy to cross ecological barriers, as all 27 autumn tracks and all 14 spring tracks of pied flycatchers included at least one full-day FLP. Another study^9^ focused on four songbird species, two *Ficedula* flycatchers and two *Acrocephalus* warblers, and reported that the most frequent strategy is non-stop flights lasting one to three days (65% of occurrence in autumn, 91% in spring), while nocturnal flights were rarely prolonged for a short time during the day while barrier crossing (5.9% of occurrence in autumn, 8.7% in spring). Clearly, in some species such as *Ficedula* flycatchers and wood warblers non-stop desert crossing is the general rule. In our study, only one Eurasian reed warbler prolonged nocturnal flights into the day in autumn, therefore most stopped before sunrise in the Sahara desert, in contrast to former work^9^ reporting prolonged flight in four of 12 autumn tracked Eurasian reed warblers (breeding birds from Germany and the Czech Republic). We sampled Eurasian reed warblers both in central Europe and in Iberia, including Andalusian birds (n = 4 out of 11) belonging to the North African short-winged *ambiguus* subspecies^15^. Northern and longer-winged birds may have a different strategy for barrier crossing and be able to perform diurnal and even non-stop flights^9^; indeed, one breeding bird from the Czech Republic revealed an autumn FLP (whereas four didn’t; Table S2), but all three Czech breeders tracked in spring displayed FLP.

During the day, increased air temperature and direct solar radiation may prevent the prolongation of nocturnal flights for most species, especially those flying at low altitude. The alternative to a non-stop flight is reducing water loss by undertaking nocturnal flights and diurnal rests^10^, especially in autumn in the absence of tailwind. Eight of ten species, however, frequently prolonged flights into the day during intermittent desert crossings. Such daylight prolongation of nocturnal flight may be adaptive, first to decrease the duration of desert crossing^9^, but also to find a suitable shaded (though shade should be available almost everywhere) or vegetated location to stop during the hottest hours to preserve the water balance and possibly refuel^16^. Large observed variances in light intensity once an individual has landed after a diurnal prolongation of a nocturnal flight may suggest use of sites with vegetation or other sources of shade, but such variance could also result from intermittent shading of the light sensor by the flight feathers of migrants performing some activity. Species-specific crossing strategies may be related to how well adapted species are to arid environments. A few studies showed differences in abundance and behavior between species at stopover sites in the desert^17,18^, mostly related to their meso- vs. xerophilic features. Interestingly, the four species with common non-stop flight strategy in spring here were reported as largely absent from stopover sites in the middle of the desert^17^. Body size might also be important to determine how well a bird can cope with high temperatures, both on the ground^19^ and in flight, with small species better able to withstand high temperatures. Their lower expected flight speeds compared to larger species might also select for diurnal flight in order to reduce the duration of barrier crossing. We must acknowledge, however, that the relative costs (for example in terms of water) of flying during the day vs. resting in an arid environment with no water and possibly no food supply, are not currently known. Understanding how this varies for birds of different body size and with different flight altitudes should be a priority for future research.

Beyond such physiological requirements and constraints, species ecology, and especially foraging behaviour, might provide some cues to explain the observed strategies. For example, foliage gleaners may be poorly adapted to diurnal stopovers in deserts because of a lack of suitable vegetation cover to forage efficiently, whereas terrestrial and aerial feeders are adapted or pre-adapted to forage in open habitats, and may also have a wider temperature tolerance and appropriate anti-predator strategies. As examples, *Ficedula* flycatchers and *Phylloscopus* and *Acrocephalus* warblers are foliage gleaners, whereas whinchats are terrestrial feeders and *Muscicapa* flycatchers are aerial feeders. A difference between *Muscicapa* and *Ficedula* flycatchers is unsurprising as spotted flycatchers neither accumulate large fat reserves in North Africa in autumn nor increase body mass along the migration route^20,21^. As a consequence, they may need to stop and refuel regularly when crossing deserts, explaining why spotted flycatchers have been observed foraging within the desert during the autumn migration^20,22^.

In addition to inter-specific differences, we also find extensive evidence of individual variation in crossing behavior, which may be driven by internal factors such as fat reserves and overall body condition, or external factors such as the occurrence of favorable vs. unfavorable winds. Birds in better condition will have larger fat reserves that decrease the need to stop and forage, as well as retaining more pectoral muscle which acts as protein stores whose metabolism would produce more water during flight if they were to continue during the day^23^.

Temperature data can provide information on the relative flying altitude while migrating over deserts, though interpreting temperature data recorded by geolocators is problematic. A simple law of thermodynamics estimates the thermal adiabatic gradient in a dry troposphere at −9.75°C per km, meaning that the air temperature decreases by approximately 1°C for every 100 m increase in altitude^24^, though the mixing of air masses means that the ideal temperature profile rarely exists in reality, so altitude estimates derived from temperature differentials between nights with migration and adjacent nights without are only approximate. Additional factors reducing accuracy include temperature differences between locations where the temperatures are recorded and the unknown extent to which body temperature and wind chill influence the temperature recorded by the logger relative to ambient temperature. Nonetheless, the observed gradient in temperature differentials (Fig. 6) should approximately reflect a relative gradient in flight altitude, for autumn and spring. A caveat is that as we used several different devices (in terms of shape and size), attached in slightly different ways to species with different plumage characteristics, influencing the amount of feather encroachment and hence insulation, temperature recorded by the tag relative to ambient temperature may not be consistent between species or deployments using different methods.

Within the observed temperature gradient, ortolan buntings in autumn have the smallest temperature differentials, so probably fly at a low altitude. The confidence interval of temperature differentials in this species includes zero in autumn, so we cannot exclude that they migrate close to ground level. Eurasian reed warblers from southwestern Europe revealed no large temperature differentials during the autumn migration, so they probably cross the desert at a low altitude too. At the other extreme, nightingales revealed the largest temperature differentials, similar to rufous-tailed scrub-robin in spring, suggesting that such large chats fly over ecological barriers at very high altitude - probably over 2000 m a.g.l.^11^.

Songbirds are reported to migrate on average at higher altitudes at night compared to daytime, and to avoid flying close to the ground in hot desert areas^25^. Migration direction and altitude can be tightly linked to wind conditions, even in small non-gliding birds^26,27^. In autumn, migrant songbirds appear to fly at low altitude over the Sahara (<1000 m a.g.l.) to benefit from favorable tailwind, despite hot and dry air increasing water loss^10^. However, most tracked songbirds migrate at higher altitudes^11^, compensating for unfavorable winds by limiting water loss by flying in humid and colder air higher up^10^. In spring, wind regimes are different^10^ and songbirds might adjust their flight altitude accordingly. Indeed, we found larger temperature differentials in spring, suggesting higher migration altitudes in spring than in autumn. Interestingly, the stopover versus non-stop flight strategies are well distributed along the temperature differential gradient, so the stop or non-stop strategies appear to be independent of flight altitude. As examples, ortolan buntings and nightingales both stop in the desert during the day and lie at either extreme of the temperature differential gradient.

Variations in altitude of barrier crossing flight bouts might also predict the risk faced by songbirds to succeed in desert crossing under future climate warming. Ground air temperatures have increased during the last decades in the Sahara^28^, and are predicted to increase by up to 7°C over the 21st century^29–31^. Recent temperature increases over the Sahara have been twice as large at ground level compared to high in the troposphere (approx. 5000 m)^28,32^, so that species flying at high elevation (such as flycatchers, chats and warblers^11^) should be less impacted than species flying close to the ground. Altogether, rising ground air temperature may considerably increase water loss for songbirds migrating at low altitude, and challenge their capacity to cross deserts, to survive their migration journey and thus to persist in a warming world. However, the high degree of variation between and within species suggests that aspects of desert crossing behaviour are plastic, so that species might be able to adjust their behavior to adapt to these new conditions.

## Material and Methods

### Study sites and species

We captured breeding ortolan bunting males on their breeding territories in Belarus, Russia (Vladimir, Belgorod and Volgograd), Lithuania, Sweden and Finland, in May-July 2012–2017^14^. We captured male and female whinchats near nests at three sites in England, Exmoor (51.1°N −3.7°W), Salisbury Plain (51.1°N −2.2°W) and Geltsdale (54.9°N −2.6°S) in 2016–2018. We captured Mediterranean flycatchers (*M*. *tyrrhenica tyrrhenica*) in northern Corsica, France, between Patrimonio and Ortiporio. This taxon is a sister species of spotted flycatcher^33^. We captured both male and female Mediterranean flycatchers near their nests in 2017 and 2018, male and female spotted flycatchers in England near nests in 2016–2019 in two regions (Devon 50.5°N −3.8°W and Cambridgeshire 52.25°N 0°E), and male wood warblers in England on territories in Devon (50.53°N −3.85°W) and the New Forest (50.8°N −1.67°W) 2016–2019. We captured tree pipits in 2016 and 2018 at two sites in England, Thetford Forest (52.34°N 0.67°E) and Forest of Dean (51.0°N 2.51°E). We captured rufous-tailed scrub-robins at Tarifa (36.02°N 5.59°W) in Spain^34^. We captured willow warblers in East Denmark (55.61°N, 12.57°E) from May to mid-June in 2014 and 2015^35^. We captured Eurasian reed warblers at four different sites, at Arroyo Ardachón, Sanlúcar la Mayor, Sevilla, in Andalucia (37.40°N 6.26°W), at Delta del Ebro, Catalunia, Spain (40.74°N 0.79°E), at Réserve de l’Estagnol in southern France (43.53°N 3.84°E), and at Mutěnice, Czech Republic (48.89°N, 17.06°E). We captured nightingales at four locations in England, in Norfolk (52.52°N 0.47°W), Suffolk (51.99°N 1.12°W, Cambridgeshire (52.3°N 0.34°W) and Kent (52.09°N 0.83°W).

### Archival light loggers

Light loggers record light intensity, which if retrieved after a migration cycle enables the calculation of the approximate position of the logger given the duration of day light and the time of solar noon. Light loggers do not transmit positions, so birds have to be recaptured and the tag retrieved to download recorded data. We used data collected by INTIGEO loggers produced by Migrate Technology: P65C2-7 loggers (0.74g) for ortolan buntings, P50Z11-7 loggers (0.50g) for Mediterranean flycatchers in 2017 and for rufous-tailed scrub-robins, P50Z11-7-DIP (0.55g) for whinchats and tree pipits, P55A1-SPLITTUBE-11DIP (0.6g) and P55Z11TOP621-11-DIP (0.7g) for nightingales, W30Z11-DIP loggers (0.32g) for spotted flycatchers (in 2016), wood warblers and willow warblers, and P30Z11-7-DIP loggers (0.36g) for spotted flycatchers (2018), Mediterranean flycatchers (2018) and Eurasian reed warblers (2017 and 2018). Loggers were attached with either UV-proof string or (stretch magic) elastic leg-loop harness to the bird’s back. The device and associated attachment material (0.02–0.04g) represented: 3.1 to 3.5% of a bird’s weight for ortolan bunting (male body mass 19–23 g during the breeding season), 3.4 to 4.3% for whinchat (body mass 14–16g), 2.6 to 4.7% in the Mediterranean flycatcher (body mass 12–14 g), 2.2 to 2.5% in the spotted flycatcher (body mass 14–16 g), 3.2 to 3.9% in the wood warbler (body mass 9–11 g), 3.5 to 4.4% in the willow warbler (body mass 8–10 g), 3.0 to 3.8% in the Eurasian reed warbler (body mass 10–13 g), 2.5 to 2.9% in the tree pipit (body mass 20–22 g), 2.8 to 3.7% for nightingales (body mass 20–22.7g) and 4.0 to 4.6% in the rufous-tailed scrub-robin (body mass 24–27 g). Overall, we retrieved 26 ortolan loggers (1 out of 10 in Belarus, 11 out of 45 in Russia, 1 out of 10 in Lithuania, 4 out of 20 in Finland, 9 out of 45 in Sweden; so 26 out of 130 equipped males, average return rate of 20%), 25 spotted flycatcher loggers from 32 returning birds (out of 80 equipped birds, returning rate 40%), 20 whinchat (from 28 returning birds, out of 110 equipped birds, return rate 26%), 5 Mediterranean flycatcher loggers for 6 returning equipped birds (3 out of 25 equipped adult birds in 2017, return rate 12%; 3 out of 10 equipped birds in 2018, return rate 30%), 3 wood warbler loggers (from 4 returning birds out of 70 equipped birds, return rate 5%), 15 willow warbler loggers (15 from 17 returning birds out of 37 equipped birds, return rate 46%)^35^, 6 Eurasian reed warblers loggers in southern Europe (out of 64 equipped birds, return rate 9%) and 5 Eurasian reed warbler loggers in the Czech Republic (out of 10 equipped males, return rate 50%), 9 tree pipit loggers (9 from 18 returning birds out of 47 equipped birds, return rate 38%), 18 nightingales (from 79 tags deployed, retrieval rate 22.8%), and 1 rufous-tailed scrub-robin logger (return rate 46%)^34^. As we retrieved some loggers after birds completed two migration cycles, in a few cases we analyzed more autumn tracks than the number of tracked individuals. On some tags, temperature was sampled by tags every 5-minutes, with the minimum and maximum values saved every 4-hour interval.

### Light intensity data and FLPs

We visually explored the light intensity data to detect FLPs (Figs. 1 and S1), as reported by previous studies^5,9^. We also identified potential prolonged flights in the day by the lower minimal temperature recorded in the morning after sunrise when a potential FLP was short (Fig. S1). When detected, we reported the date (Table S2), and the light intensity and temperature (examples shown in Fig. 1). To estimate the duration of prolonged flight during daylight, we defined sunrise as the first hour when the recorded light intensity was >2. We then considered the hour of the abrupt drop in light intensity as the landing time, and deduced the daylight flight duration by comparison to the sunrise time.

To obtain statistics on the proportion of birds showing a FLP, and the proportional frequency of individuals flying during hour intervals during the day, and on proportional frequency of landing time, we considered that migrant birds crossing a desert can potentially display up to three days with FLP (which is the maximum observed here, though desert crossing could be shorter than three days and nights). For example, the 28 autumn migration tracks for ortolan buntings could generate 28*3 = 84 FLP.

According to the information reported in Table S2, we considered in Fig. 3 that individuals showing two full-day FLPs have crossed the desert without stopping, and that individuals showing a full-day FLP followed (or not) by an incomplete FLP in the next day probably did so too – so we considered that these individuals did not stop over in the desert. Indeed, a non-stop flight of 36 hours at a 50 km.h-1 speed, attainable by the species considered here with moderate wind assistance, is sufficient to overfly 1800 km of desert.

We tested if the season and the date of barrier crossing (as the first observed FLP; see Table S1) within the season could predict the probability to perform a non-stop flight over the desert (see Methods for details). To do so, we performed a generalized linear model (binomial distribution, chi-square test), first in a model accounting for species and season (n = 216), then including date as a further predictor (known only for individuals with a detected FLP, n = 165).

### Temperature data

A first exploration of temperature data consisted of a visual check of temperatures during possible autumn and spring barrier crossings (1 August–30 November and 1 March–15 May) in order to detect relative temperature anomalies lasting one to four days (Fig. S2). Temperature data recorded may be correlated with weather, but can also indicate potential changes in altitude, especially when birds fly at high altitude. We expected low minimal and/or maximal temperatures recorded during a migration flight bout if it lasted during the four preceding hours (temperature data being stored every four hours). After identifying potential consecutive nights with relatively low temperatures in a long time series (see Fig. S2 for examples), we checked for the pattern of light data of the same days, and estimated the stationary area of each individual before and after the presumed barrier crossing migration flight.

We estimated temperature differentials between days with and without prolonged nocturnal flights, as large differentials might reveal higher than usual flight altitudes. We compared the morning or noon temperatures (as recorded within four hours after sunrise for part-day FLP with an abrupt ending, or at noon for full-day FLP) recorded on a day of prolonged nocturnal flight to the temperature at the same hour the previous day, or the next day that occurred without a prolonged flight during daylight.

We estimated differences in temperatures between the days before and after barrier crossing flights to verify that the differences were not too sensitive to regional weather differences (for ortolan buntings, the species with most available data, generalized linear model, comparison of temperature differentials recorded with the day before vs after a diurnal flight prolongation, t_51_ = 0.007, P > 0.99; see Fig. S3). If an individual prolonged a nocturnal flight for a short period after sunrise, the minimal temperature recorded during the next 4 hours should refer to the temperature measured while flying at a relatively higher altitude, as the temperature in the air should be lower than on the ground. When a part-day FLP was detected, we reported the minimal temperature recorded by the logger within the first four hours after sunrise for days with FLP, and the same temperature recorded for the same time period during the first previous or the first next day without FLP, and calculated the differential between these two temperatures. As an example, in ortolan buntings, the time between the minimal temperature recorded and sunrise was on (average ± s.d.) 91 ± 35 minutes (range = 20–210 minutes) in autumn, 120 ± 59 minutes (range = 22–305 minutes) in spring. For days with full-day FLP, we estimated the temperature differential with the previous and/or next day around noon. For individuals with no detected FLP but obvious nocturnal flights detected from very low temperatures, we compared the minimum temperature recorded by the logger between 11pm and 2am (around midnight) and compared it to the first previous and next night with no flight bout.

### Estimating stationary areas

We used different approaches to estimate coordinates of stationary areas used before and after detected FLP days, according to species. For willow warblers, we reported stationary areas published in a previous study for the same individuals^34^. For Mediterranean flycatchers, we estimated stationary areas as periods of stable latitude and longitude estimates as calculated with GeoLight v2.0 with default parameters, including a −6° sun elevation angle. For ortolan buntings, we used the GeoLight v2.0.1 package to estimate twilight times using a threshold method^13^. We used the ‘LoessFilter’ function to validate twilight events and to identify and remove those influenced by shading events at dawn and dusk. For English spotted flycatchers and wood warblers, we used on-bird in-habitat calibration during breeding in the deployment year as the method of estimating the sun elevation angle. We used the ‘getElevation’ function to calculate SEAs, using the zero elevation angle of the twilight error distribution. We then used the ‘changeLight’ and ‘mergesites2’ functions with a gamma error distribution to estimate stationary periods and locations. For ortolan buntings, when the effect of equinox was strong, resulting in high latitudinal variation, we calculated the latitudinal mode (not the median) when latitude varied little. For Eurasian reed warblers, we annotated twilight events in TwGeos and conducted positioning in GeoLight v2.0.1. We used sun elevation angles from on-bird in-habitat calibration during breeding in the deployment year for estimating positions before the barrier crossing and sun elevation angles from Hill-Ekstrom calibration of merged wintering stationary periods for estimating positions after the barrier crossing. For ortolan buntings, when the effect of equinox was strong, resulting in high latitudinal variation, we calculated the latitudinal mode (not the median) when latitude varied little^14^. We did not estimate stationary areas for whinchats, tree pipits and nightingales.

### Legal and ethical statements

All captures and tagging in the United Kingdom were licensed by the Special Marks Technical Panel operating on behalf of the British Trust for Ornithology and the UK Government’s Home Office. All captures and tagging in France were licensed by the Centre de Recherches sur la Biologie des Populations d’Oiseaux. Captures and tagging of ortolan Buntings and Eurasian reed warblers in Catalonia were licensed by the Generalitat de Catalunya (Catalan Ringing Scheme) and with local permission of the Consorci per a la Protecció I la Gestió dels Espais Naturals del delta del Llobregat, and the Parc Natural del Delta de l’Ebre. Captures and tagging of reed warblers in Andalusia were licensed by the Andalusian Regional Government and Aranzadi Ringing Scheme. Captures and tagging of scrub-robins in Spain was undergone under the approved authorization from the Spanish Ministry of Agriculture and Fisheries, Food and Environment, and the regional government of Andalucia. Captures of reed warblers in the Czech Republic were conducted under a ringing licence from the Czech Bird Ringing Scheme (ringing licence number 906); geolocator attachment was approved by the Municipality of Hodonín (permit number MUHOCJ 17666/2018/OŽP). Captures and tagging of ortolan buntings in Russia (Vladimir Region) were undergone under the approved authorization from the Bird Ringing Centre of Russia (Moscow) and the full support of the Centre that provided the metal rings ‘Moskwa’ used. Captures and tagging of ortolan buntings in Belgorod and Volgograd, Russia, were undergone under the approved authorization of the Belogorie Nature Reserve and the Volga-Ahtubinskaya flood plain Natural Park, respectively. In Belarus, captures and tagging were undergone under the approval of the Department of Biological Sciences of the National Academy of Sciences. In Lithuania, captures and tagging were authorized by the Lithuanian Bird Ringing Centre, at the Zoological Museum in Kaunas. For Poland, captures and tagging were licensed by the Gdańsk Ringing Scheme at the Museum and Institute of Zoology of the Polish Academy of Sciences. In Sweden, captures and tagging were licensed by the Swedish Bird Ringing Centre located at the Museum of Natural History in Stockholm. The licenses secure species knowledge and ethic handling when ringing. Captures and tagging of ortolan buntings in Finland was undergone under the approved authorization from the Centres for Economic Development, Transport and the Environment (ELY Centres). In Denmark, tagging of willow warblers was approved by the Copenhagen Bird Ringing Centre (SN302–009) under permission from the Danish Forest and Nature Agency. In all countries, captures and tagging were carried out in accordance with national ethical guidelines.

## Supplementary information

Supporting Information.

## Data Availability

Data used in this study is available upon request to the corresponding author.
