# Supplementary material for: Desert crossing strategies of migrant songbirds vary between and within species
Source: Sci Rep. 2019 Dec 27;9:20248. doi: 10.1038/s41598-019-56677-4 (PMC6934701; doi:10.1038/s41598-019-56677-4)
Supplement: Supplementary file 1 — Supporting Information. [file 41598_2019_56677_MOESM1_ESM.docx]

**Supplementary Materials for**

**Desert crossing strategies of migrant songbirds vary between and within species**

Frédéric Jiguet, Malcolm Burgess, Kasper Thorup, Greg Conway, José Luis Arroyo Matos, Lee Barber, John Black, Niall Burton, Joan Castelló, Gary Clewley, José Luis Copete, Michel Alexandre Czajkowski, Svein Dale, Tony Davis, Valery Dombrovski, Mike Drew, Jaanus Elts, Vicky Gilson, Emilienne Grzegorczyk, Ian Henderson, Michael Holdsworth, Rob Husbands, Romain Lorrilliere, Riho Marja, Simonas Minkevicius, Caroline Moussy, Peter Olsson, Alejandro Onrubia, Marc Pérez, Joseph Piacentini, Markus Piha, Jean-Marc Pons, Petr Procházka, Marko Raković, Harriet Robins, Tuomas Seimola, Gunnar Selstam, Michał Skierczyński, Jan Sondell, Jean-Claude Thibault, Anders P. Tøttrup, Justin Walker, Chris Hewson

**Appendix 1. Species-specific details of the detected FLPs, including information on average time spent in flight after sunrise if occurring**

We detected a FLP in 25 of the 28 autumn (89%) and in 20 of the 24 spring (83%) recorded barrier crossings for ortolan bunting, which occurred in one to three consecutive days (Fig. 1; Table S2). In autumn, an FLP was detected for one day for 18 individuals (64% of all individuals), two days for 5 individuals (18%) and three days for 2 individuals (11%). The remaining 10% did not show any FLP. The average time spent in flight during daylight was 341 minutes after sunrise (± 148 s.d., median 335 min, range 80-655 min; n=34), with an increasing number of individuals landing as the time progressed (Fig. 3a). We detected no FLP lasting a complete day in autumn. In spring, FLP was detected for one day for 13 individuals (54% of all individuals), two days for 5 individuals (21%) and 3 days for 2 individuals (8%). The remaining 17% showed no FLP. The time spent in flight during the daylight was 396 minutes after sunrise (± 227 s.d., median 370 min, range 95-805, n=29; Fig. 3b). FLP during a complete day was detected for 5 individuals and only in spring, representing 21% of individuals and 25% of the spring detected FLP (see Fig. 2). Such individuals are not reported as having landed in Fig. 3.

For whinchats, we detected a FLP in 11 of the 20 individuals tracked in autumn, and in all 16 individuals tracked in spring. In case of a partial diurnal prolongation of a nocturnal flight, the time spent in flight during the daylight was 425 minutes after sunrise (± 160 s.d., median 440 min, range 105-610, n=10) in autumn, 439 minutes in spring (± 110 s.d., median 440 min, range 185-620, n=14).

We detected a FLP in 19 of the 24 autumn tracks of spotted flycatchers: for one day in 11 individuals (one individual not landing), for two days in 7 individuals (two not landing during the first day), for three days in 1 individual (the first day without landing). When prolonged and if not lasting the complete day, the diurnal flight ended by landing 330 minutes after sunrise (± 179 s.d., median 280 min, range 120-715 min, n=23; Fig. 3c). In spring, the quality of light intensity recording was sufficient to study 14 individuals, and all but one performed diurnal flights, during one day (2 individuals, with no landing), two days (10 individuals, 7 of them with no landing on the first day, 2 of them with no landing during the two days) or three days (1 individual, with no landing on the second day). We detected a FLP during at least one complete day for 12 individuals, representing 86% of the individuals and 56% of the detected FLP. If occurring, diurnal landing occurred after 479 min (± 147 s.d., median 510 min, range 120-655, n=11; Fig. 3d).

We detected a full light pattern for all 5 tracked Mediterranean flycatchers in autumn (for one day in 2 individuals, for two days in 3 individuals), with landings 268 minutes after sunrise (± 177 s.d., median 242 min, range 75-575 min; n=8), and also in spring for the two loggers which were still recording (Table S2 Fig. 2), with one individual performing a non-stop flight during a complete day.

We detected FLP for 11 of the 15 tracked willow warblers (for one day in 9 individuals, for two days in two individual), with a FLP during a complete day for 4 individuals (Table S1, Fig. 1). The other 4 willow warblers did not prolong nocturnal flights into the day. When prolonged and if not lasting the complete day, the diurnal flight ended by landing 324 ± 122 minutes after sunrise (median 290 min, range 210-510, n=8; Fig. 2d).

Wood warblers displayed very similar patterns in autumn and spring, with two successive FLPs through a complete day, with one individual landing just before sunset on the second day in spring (Fig. 2).

We detected a FLP for only one of the six Eurasian reed warblers in autumn, but for all 3 individuals tracked in spring. All of them have migrated over the Sahara (Table S2, Fig. 4).

We detected a FLP for 8 of 9 tracked tree pipits, during two successive days in autumn (with a first full light pattern lasting a complete day in 2 individuals, two complete successive days in 1 individual). In spring, we detected FLP for the 8 tracked individuals with available data (5 individuals with two successive FLPs, including during a complete first day for 4 individuals, and 3 individuals with FLP in one day). When prolonged and if not lasting the complete day, the diurnal flight ended by landing 351 ± 93 minutes after sunrise in autumn (median 360 min, range 205-535, n=11), and 330 ± 152 minutes after sunrise in spring (median 365 min, range 85-5605, n=9).

The rufous-tailed robin probably prolonged a nocturnal flight into one day for a very short time before landing (abrupt drop in light intensity), but not on the two subsequent days with nocturnal flight bouts identified by low temperature records (Fig. 1, Fig. S2). In spring, the same bird prolonged its nocturnal flight for a short time on one day, as revealed by low minimal temperatures recorded after sunrise (Table S2, Fig. 2). Desert crossing during early May was further confirmed by the periods and locations of the previous and subsequent stationary area (see Fig. 7 for locations).

We detected a FLP for 10 of 20 tracks of the 18 nightingales in autumn (two individuals tracked during more than one year), during one single day (only one lasting a complete day), and for 7 of the 10 nightingales tracks in spring, for only one day in six cases, for two days in one case. When prolonged and if not lasting the complete day (so excluding one case), the diurnal flight ended by landing 257 minutes after sunrise in autumn (± 125 s.d., median 185 min, range 155-510, n=9), and 395 minutes after sunrise in spring (± 162 s.d., median 475 min, range 140-600, n=7).

Table S1. First and last dates with detected FLP (e.g. prolonged nocturnal flight into the day) while desert crossing, by season and for species with at least three tracked individuals. Note that ortolan buntings crossed the Arabian desert two weeks earlier than the Sahara in autumn but at similar dates in spring.

| Season | Species | First date | Last date |
| --- | --- | --- | --- |
| Autumn | ortolan bunting (Sahara) | 26 Sep | 9 Oct |
|  | ortolan bunting (Arabia) | 12 Sep | 29 Sep |
|  | whinchat | 19 Sep | 4 Oct |
|  | spotted flycatcher | 6 Sep | 1 Oct |
|  | Mediterranean flycatcher | 19 Sep | 6 Oct |
|  | willow warbler | 1 Sep | 5 Oct |
|  | wood warbler | 28 Aug | 3 Sep |
|  | tree pipit | 21 Sep | 17 Oct |
|  | nightingale | 10 Sep | 3 Oct |
| Spring | ortolan bunting (Sahara) | 3 Apr | 18 Apr |
|  | ortolan bunting (Arabia) | 9 Apr | 16 Apr |
|  | whinchat | 1 Apr | 1 May |
|  | spotted flycatcher | 17 Apr | 7 May |
|  | Eurasian reed warbler | 15 Apr | 27 Apr |
|  | tree pipit | 18 Mar | 9 Apr |
|  | nightingale | 14 Mar | 23 Apr |

Table S2. Tracked individuals, with references to logger number (* if a second tracking year), species, breeding country, desert crossed during migration and dates with FLP(s) (in bold if a full-day FLP). If no information is reported for the spring migration, this is because the battery of the logger was dead.

| Individual | Species | Country | Desert | Date(s) of Full Light Pattern |
| --- | --- | --- | --- | --- |
| B730 | Ortolan Bunting | Sweden | Sahara | 9 Oct 2012 |
|  |  |  |  | 17-18 Apr 2013 |
| B734 | Ortolan Bunting | Sweden | Sahara | 9 Oct 2012 |
|  |  |  |  | **6** **Apr** 2013 |
| B736 | Ortolan Bunting | Sweden | Sahara | No FLP (autumn 2012) |
|  |  |  |  | 6 Apr 2013 |
| F170 | Ortolan Bunting | Sweden | Sahara | 8 Oct 2013 |
|  |  |  |  | **16** **Apr** 2014 |
| F173 | Ortolan Bunting | Sweden | Sahara | 6 Oct 2013 |
|  |  |  |  | 5-6 Apr 2014 |
|  |  |  |  | 29 Sep 2014* |
| F178 | Ortolan Bunting | Sweden | Sahara | No FLP (autumn 2013) |
|  |  |  |  | 10-11-12 Apr 2014 |
| F184 | Ortolan Bunting | Sweden | Sahara | 6 Oct 2013 |
|  |  |  |  | **9** **Apr** 2014 |
| F191 | Ortolan Bunting | Sweden | Sahara | 7 Oct 2013 |
|  |  |  |  | Logger dead (spring) |
| F196 | Ortolan Bunting | Sweden | Sahara | 30 Sep 2013 |
|  |  |  |  | 4-5 Apr 2014 |
|  |  |  |  | 26 Sep 2014* |
| R231 | Ortolan Bunting | Finland | Sahara | 4 Oct 2015 |
|  |  |  |  | **9** **Apr** 2016 |
| R234 | Ortolan Bunting | Finland | Sahara | 9 Oct 2015 |
|  |  |  |  | **6** **Apr** 2016 |
| R240 | Ortolan Bunting | Finland | Sahara | 9 Oct 2015 |
|  |  |  |  | 8-9-10 Apr 2016 |
| R262 | Ortolan Bunting | Finland | Sahara | 6 & 9 Oct 2016 |
|  |  |  |  | 11 Apr 2017 |
| R024 | Ortolan Bunting | Lithuania | Sahara | 28 Sep 2015 |
|  |  |  |  | 3 Apr 2016 |
| R007 | Ortolan Bunting | Belarus | Arabia | 26-27 Sep 2015 |
|  |  |  |  | 11 Apr 2016 |
| R284 | Ortolan Bunting | Russia | Arabia | 29 Sep 2016 |
| R285 | Ortolan Bunting | Russia | Arabia | 18-19 Sep 2016 |
|  |  |  |  | 13 Apr 2017 |
| R308 | Ortolan Bunting | Russia | Arabia | 23-24 Sep 2015 |
|  |  |  |  | No FLP (spring) |
| R313 | Ortolan Bunting | Russia | Arabia | 24 Sep 2016 |
|  |  |  |  | 9-10 Apr 2017 |
| R328 | Ortolan Bunting | Russia | Arabia | 18-19-20 Sep 2015 |
|  |  |  |  | 15-16 Apr 2016 |
| R334 | Ortolan Bunting | Russia | Arabia | 17 Sep 2015 |
|  |  |  |  | No FLP (spring) |
| R342 | Ortolan Bunting | Russia | Arabia | No FLP (autumn) |
|  |  |  |  | 9 Apr 2016 |
| R345 | Ortolan Bunting | Russia | Arabia | 12 & 14 Sep 2015 |
|  |  |  |  | 15 Apr 2016 |
| R348 | Ortolan Bunting | Russia | Arabia | 17-18-19 Sep 2015 |
|  |  |  |  | No FLP (spring) |
| R353 | Ortolan Bunting | Russia | Arabia | 23 & 25 Sep 2015 |
|  |  |  |  | No FLP (spring) |
| R364 | Ortolan Bunting | Russia | Arabia | 19 Sep 2015 |
|  |  |  |  | 13 Apr 2016 |
| BA279 | Whinchat | England | Sahara | 27 Sep 2016 |
|  |  |  |  | **1-**2 **Apr** 2017 |
| BA282 | Whinchat | England | Sahara | No FLP (autumn) |
|  |  |  |  | **17-**18 **Apr** 2017 |
| BA285 | Whinchat | England | Sahara | No FLP (autumn) |
|  |  |  |  | 5 Apr 2017 |
| BA287 | Whinchat | England | Sahara | **27 Sep** 2016 |
|  |  |  |  | **22** **Apr** 2017 |
| BA288 | Whinchat | England | Sahara | No FLP (autumn) |
|  |  |  |  | **3-4** **Apr** 2017 |
| BA289 | Whinchat | England | Sahara | No FLP (autumn) |
|  |  |  |  | **15 Apr** 2017 |
| BA294 | Whinchat | England | Sahara | No FLP (autumn) |
|  |  |  |  | **9 Apr** 2017 |
| BA296 | Whinchat | England | Sahara | 27 Sep 2016 |
| BA921 | Whinchat | England | Sahara | No FLP (autumn) |
|  |  |  |  | **30 Apr**-1 May 2017 |
| BA923 | Whinchat | England | Sahara | No FLP (autumn) |
|  |  |  |  | 16-17 Apr 2017 |
| BG247 | Whinchat | England | Sahara | 19 Sep 2017 |
|  |  |  |  | **13**-14 **Apr** 2018 |
| BG251 | Whinchat | England | Sahara | 2-4 Oct 2017 |
|  |  |  |  | 18 Apr 2018 |
| BG404 | Whinchat | England | Sahara | **24 Sep** 2017 |
| BG410 | Whinchat | England | Sahara | No FLP (autumn) |
|  |  |  |  | **20**-21 **Apr** 2018 |
| BG415 | Whinchat | England | Sahara | **27 Sep** 2017 |
|  |  |  |  | **12-**13-14 **Apr** 2018 |
| BG416 | Whinchat | England | Sahara | 2-4 Oct 2017 |
|  |  |  |  | **12-13 Apr** 2018 |
| BG419 | Whinchat | England | Sahara | 2 Oct 2017 |
|  |  |  |  | **30 Apr-1 May** 2018 |
| BG430 | Whinchat | England | Sahara | No FLP (autumn) |
|  |  |  |  | 27-28 Apr 2018 |
| BG431 | Whinchat | England | Sahara | 2 Oct 2017 |
|  |  |  |  | **14-**16 **Apr** 2018 |
| BG459 | Whinchat | England | Sahara | 30 Sep 2017 |
| BA176 | Spotted Flycatcher | England | Sahara | 15 Sep 2016 |
| BA181 | Spotted Flycatcher | England | Sahara | 30 Sep 2016 |
| BA185 | Spotted Flycatcher | England | Sahara | 10 Sep 2016 |
| BA202 | Spotted Flycatcher | England | Sahara | 19 Sep 2016 |
| BA209 | Spotted Flycatcher | England | Sahara | 26 Sep 2016 |
| BA210 | Spotted Flycatcher | England | Sahara | 30 Sep-1 Oct 2016 |
| BA385 | Spotted Flycatcher | England | Sahara | 20-21 Sep 2016 |
|  |  |  |  | 2-3 May 2017 |
| BA402 | Spotted Flycatcher | England | Sahara | **6**-7-8 **Sep** 2016 |
|  |  |  |  | **17-18** **Apr** 2017 |
| BA406 | Spotted Flycatcher | England | Sahara | **28**-29 **Sep** 2016 |
| BA410 | Spotted Flycatcher | England | Sahara | 27 Sep 2016 |
|  |  |  |  | **30** **Apr** 2017 |
| BA411 | Spotted Flycatcher | England | Sahara | No FLP (autumn) |
|  |  |  |  | **2**-3 **May** 2017 |
| BA422 | Spotted Flycatcher | England | Sahara | 21-22 Sep 2016 |
|  |  |  |  | **6**-7 **May** 2017 |
| BN265 | Spotted Flycatcher | England | Sahara | 16-17 Sep 2018 |
|  |  |  |  | No FLP (spring) |
| BN269 | Spotted Flycatcher | England | Sahara | No FLP (autumn) |
|  |  |  |  | **6-**7 **May** 2019 |
| BN271 | Spotted Flycatcher | England | Sahara | No FLP (autumn) |
| BN274 | Spotted Flycatcher | England | Sahara | No FLP (autumn) |
|  |  |  |  | **3**-4 **May** 2019 |
| BN275 | Spotted Flycatcher | England | Sahara | 15-16 Sep 2018 |
|  |  |  |  | **24**-25 **Apr** 2019 |
| BN276 | Spotted Flycatcher | England | Sahara | 17 Sep 2019 |
| BN280 | Spotted Flycatcher | England | Sahara | 24 Sep 2019 |
|  |  |  |  | **5**-6 **May** 2019 |
| BN309 | Spotted Flycatcher | England | Sahara | 23 Sep 2019 |
|  |  |  |  | 29-**30** **Apr**-1May 2019 |
| BN312 | Spotted Flycatcher | England | Sahara | 11-**12** **Sep** 2019 |
|  |  |  |  | **24-25** **Apr** 2019 |
| BN315 | Spotted Flycatcher | England | Sahara | **11** **Sep** 2018 |
|  |  |  |  | **1** **May** 2019 |
| BN320 | Spotted Flycatcher | England | Sahara | 24 Sep 2018 |
|  |  |  |  | 5-6 May 2019 |
| BN324 | Spotted Flycatcher | England | Sahara | No FLP |
| BD454 | Mediterranean Flycatcher | Corsica | Sahara | 27 Sep 2017 |
|  |  |  |  | **30** **Apr**-1 May 2018 |
| BD455 | Mediterranean Flycatcher | Corsica | Sahara | 6 Oct 2017 |
|  |  |  |  | 7-8 May 2018 |
| BD465 | Mediterranean Flycatcher | Corsica | Sahara | 30 Sep & 2 Oct 2017 |
| BL115 | Mediterranean Flycatcher | Corsica | Sahara | 19-20 Sep 2018 |
| BL958 | Mediterranean Flycatcher | Corsica | Sahara | 27-28 Sep 2018 |
| L898 | Willow Warbler | Denmark | Sahara | 25 Sep 2014 |
| L904 | Willow Warbler | Denmark | Sahara | No FLP |
| L906 | Willow Warbler | Denmark | Sahara | **27 Sep** 2014 |
| L935 | Willow Warbler | Denmark | Sahara | **1-**2 **Sep** 2014 |
| L961 | Willow Warbler | Denmark | Sahara | 15 Sep 2014 |
| L965 | Willow Warbler | Denmark | Sahara | 26 Sep 2014 |
| L967 | Willow Warbler | Denmark | Sahara | 5 Oct 2014 |
| L974 | Willow Warbler | Denmark | Sahara | No FLP |
| L981 | Willow Warbler | Denmark | Sahara | No FLP |
| U023 | Willow Warbler | Denmark | Sahara | 18 Sep 2015 |
| U024 | Willow Warbler | Denmark | Sahara | 10 Sep 2015 |
| U031 | Willow Warbler | Denmark | Sahara | No FLP |
| U032 | Willow Warbler | Denmark | Sahara | **9-**10 **Sep** 2015 |
| U033 | Willow Warbler | Denmark | Sahara | **20** **Sep** 2015 |
| U037 | Willow Warbler | Denmark | Sahara | 19 Sep 2015 |
| BA204 | Wood Warbler | England | Sahara | **3-4** **Sep** 2016 |
| BA424 | Wood Warbler | England | Sahara | **2-3** **Sep** 2016 |
|  |  |  |  | **24**-25 **Apr** 2017 |
| BM370 | Wood Warbler | England | Sahara | **28-29** **Aug** 2018 |
|  |  |  |  | **7**-8 **Apr** 2019 |
| BD505 | Eurasian Reed Warbler | Spain | Sahara | No FLP (autumn) |
| BD508 | Eurasian Reed Warbler | Spain | Sahara | No FLP (autumn) |
| BD541 | Eurasian Reed Warbler | France | Sahara | No FLP (autumn) |
| BL945 | Eurasian Reed Warbler | Spain | Sahara | No FLP (autumn) |
| BL947 | Eurasian Reed Warbler | Spain | Sahara | No FLP (autumn) |
| BL955 | Eurasian Reed Warbler | Spain | Sahara | No FLP (autumn) |
| BP347 | Eurasian Reed Warbler | Czech Rep. | Sahara | No FLP (autumn) |
|  |  |  |  | 21-22 Apr 2019 |
| BP349 | Eurasian Reed Warbler | Czech Rep. | Sahara | No FLP (autumn) |
|  |  |  |  | 27 Apr 2019 |
| BP352 | Eurasian Reed Warbler | Czech Rep. | Sahara | No FLP (autumn) |
| BP354 | Eurasian Reed Warbler | Czech Rep. | Sahara | 2 Sep 2018 |
|  |  |  |  | 15 Apr 2019 |
| BP355 | Eurasian Reed Warbler | Czech Rep. | Sahara | No FLP (autumn) |
| BL907 | Tree Pipit | England | Sahara | 29-30 Sept 2018 |
|  |  |  |  | 9 Apr 2019 |
| BL910 | Tree Pipit | England | Sahara | 16-17 Oct 2018 |
|  |  |  |  | **21**-22 **Mar** 2019 |
| BL909 | Tree Pipit | England | Sahara | 21-22 Sep 2018 |
|  |  |  |  | 29-30 Mar 2019 |
| BL919 | Tree Pipit | England | Sahara | No FLP |
|  |  |  |  | **18**-19 **Mar** 2019 |
| BL928 | Tree Pipit | England | Sahara | **30 Sep-1 Oct** 2018 |
|  |  |  |  | **7-**8 **Apr** 2019 |
| Z808 | Tree Pipit | England | Sahara | 26-27 Sep 2016 |
|  |  |  |  | **23**-24 **Mar** 2017 |
| Z812 | Tree Pipit | England | Sahara | **26**-27 **Sep** 2016 |
|  |  |  |  | 27 Mar 2017 |
| Z820 | Tree Pipit | England | Sahara | 27-28 Sep 2016 |
| Z822 | Tree Pipit | England | Sahara | **2**-3 **Oct** 2016 |
|  |  |  |  | 22 Mar 2017 |
| L998 | Rufous-tailed Scrub-Robin | Spain | Sahara | 11 Sep 2015 |
|  |  |  |  | 3 May 2016 |
| BE482 | Nightingale | England | Sahara | No FLP (autumn) |
|  |  |  |  | 4 Apr 2018 |
| BE491 | Nightingale | England | Sahara | 28 Sep 2017 |
|  |  |  |  | No FLP (spring) |
| BE497 | Nightingale | England | Sahara | 11 Sep 2017 |
|  |  |  |  | 27-28 Mar 2018 |
|  |  |  |  | 19 Sep 2018* |
| BE506 | Nightingale | England | Sahara | 16 Sep 2017 |
|  |  |  |  | 23 Apr 2018 |
| BE509 | Nightingale | England | Sahara | 10 Sep 2017 |
|  |  |  |  | 25 Mar 2018 |
|  |  |  |  | 15 Sep 2018* |
| BE521 | Nightingale | England | Sahara | No FLP (autumn) |
|  |  |  |  | 14 Mar 2018 |
| S410 | Nightingale | England | Sahara | **3 Oct** 2017 |
| S412 | Nightingale | England | Sahara | No FLP (autumn) |
| S413 | Nightingale | England | Sahara | No FLP (autumn) |
| S414 | Nightingale | England | Sahara | No FLP (autumn) |
| S418 | Nightingale | England | Sahara | No FLP (autumn) |
| S420 | Nightingale | England | Sahara | 20 Sep 2015 |
| S421 | Nightingale | England | Sahara | No FLP (autumn) |
| S422 | Nightingale | England | Sahara | No FLP (autumn) |
| Z401 | Nightingale | England | Sahara | 16 Sep 2016 |
|  |  |  |  | No FLP (spring) |
| Z408 | Nightingale | England | Sahara | No FLP (autumn) |
|  |  |  |  | No FLP (spring) |
| Z411 | Nightingale | England | Sahara | No FLP (autumn) |
|  |  |  |  | 2 Apr 2017 |
| Z412 | Nightingale | England | Sahara | 3 Oct 2016 |
|  |  |  |  | 18 Mar 2017 |

Figure S1. Examples of light intensity and temperature patterns outside desert crossing, illustrating how FLP is determined by visualising graphic representation of the light data.

R240, Ortolan Bunting, 2-11 July 2015. Light intensity is high during the whole day and highly variable from 2 to 6 July, then becomes on average lower and peaking at noon from 7 July onwards; this change might correspond to a switch from intensive territory defense (and long times spent singing at top of bushes or trees) to chick feeding after putative egg hatching on 7 July (time mainly spent foraging in the vegetation). Note also the very short dark times at nights.

R240, Ortolan Bunting, 25 January-3 February 2016. The bird is on its wintering grounds, is more exposed to light in early morning and late afternoon, less exposed at noon when it probably avoids hot temperature by hiding in vegetation.

R240, Ortolan Bunting, 1-20 October 2015. A long series of 20 full days, a FLP on 9 October morning, corresponding to a drop in minimal temperature that morning. The nights from 7 to 8 and 8 to 9 October have particularly low minimal and maximal temperatures, corresponding to flight migration bouts at some altitude.

R348, Ortolan Bunting, 1-30 September 2015. A long series of 30 full days, with obvious FLP on 17 and 18 September, with a very short one on 19 September, the diurnal prolongation of the flight being confirmed by a low early morning minimal temperature. The very low minimal temperature during the night from 13 to 14 September might correspond to a nocturnal migration bout at high altitude, but without diurnal prolongation.


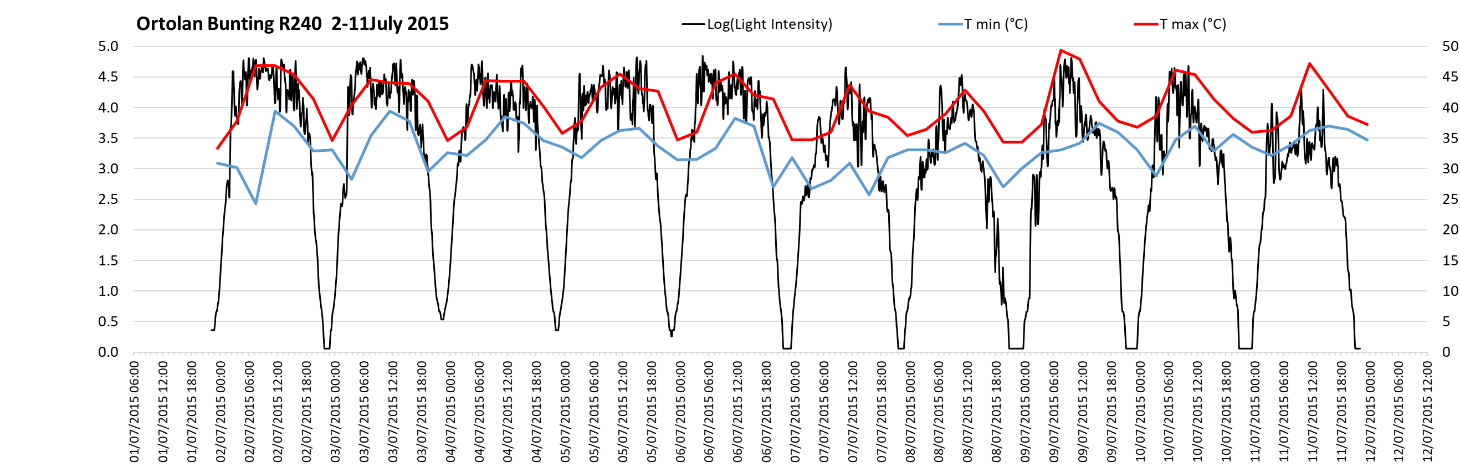


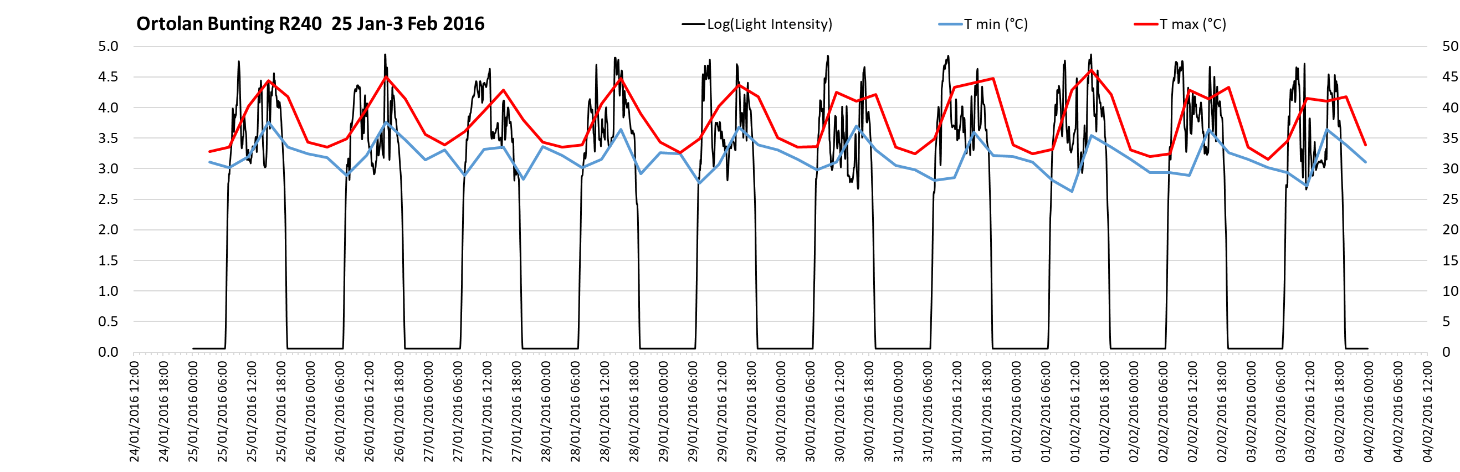


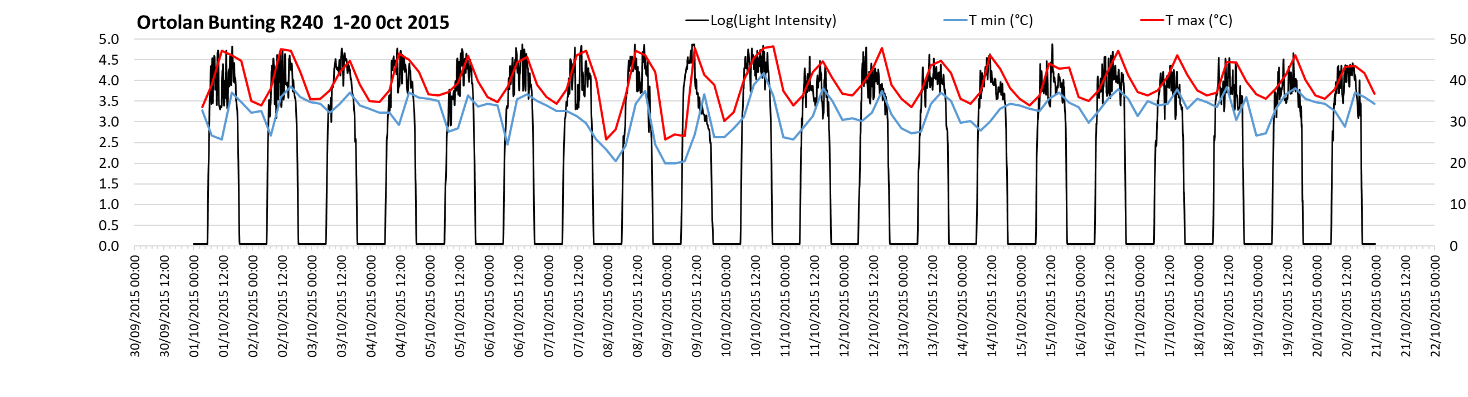

Figure S2. Long time series of temperature and humidity records obtained from loggers.

BD454, fitted on a Mediterranean Flycatcher, first 1 August-30 November 2017, reporting minimal temperature (blue), maximal temperature (red, scale on the left), and air humidity (black, scale on the right); then same logger 15 September-15 October (minimal and maximal temperatures). Checking temperature and humidity data during 4 months illustrates the regularity of records, and the potential link between temperature variability and weather (air humidity) in late October and early November. The largest drops in nocturnal minimal and maximal temperatures occurred at the end of September (26-27, 28-29, 29-30 Sept and 30 Sept-1 Oct), corresponding to four nights of flight migration bouts at high altitude. Light intensity patterns of these days are reported in Fig. 1. The third panel illustrates how we estimated differences in minimal temperatures between nights or mornings with migration flights or on ground.

R313, fitted on an Ortolan Bunting, 1 March-15 May 2017, reporting minimal temperature, maximal temperature and air humidity. The full light pattern revealed a desert crossing on 9-10 April (see Fig. 1), and temperature dropped during the nights of these two dates. At the end of April the bird arrived on its breeding grounds.

L998, fitted on a rufous-tailed scrub-robin, 1 August – 30 November. There are several short periods of temperature drops, but only one is outside a humid event, in early September, with the lowest minimal and maximal temperatures recorded during the four months – the srub-robin crossed the desert during these days.


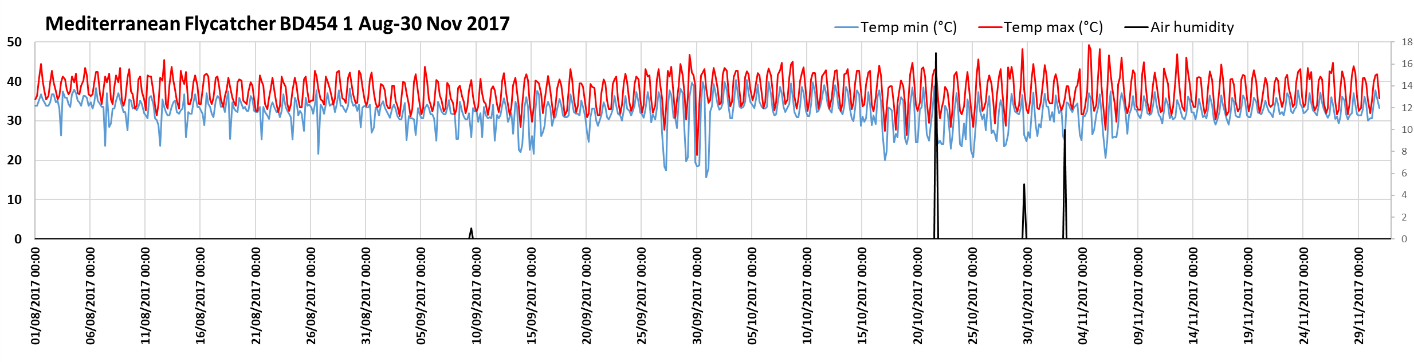


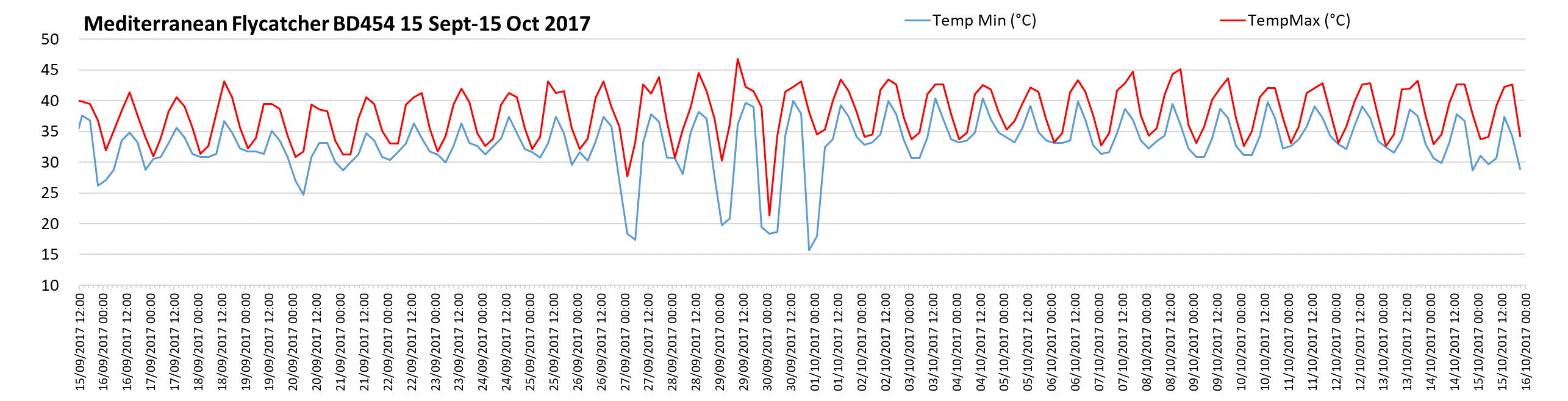


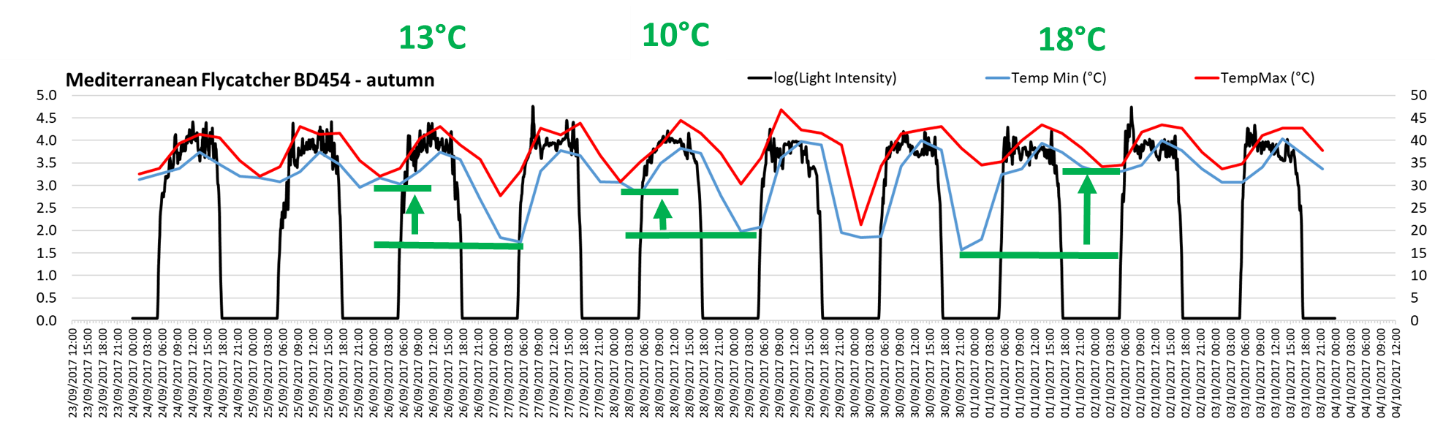


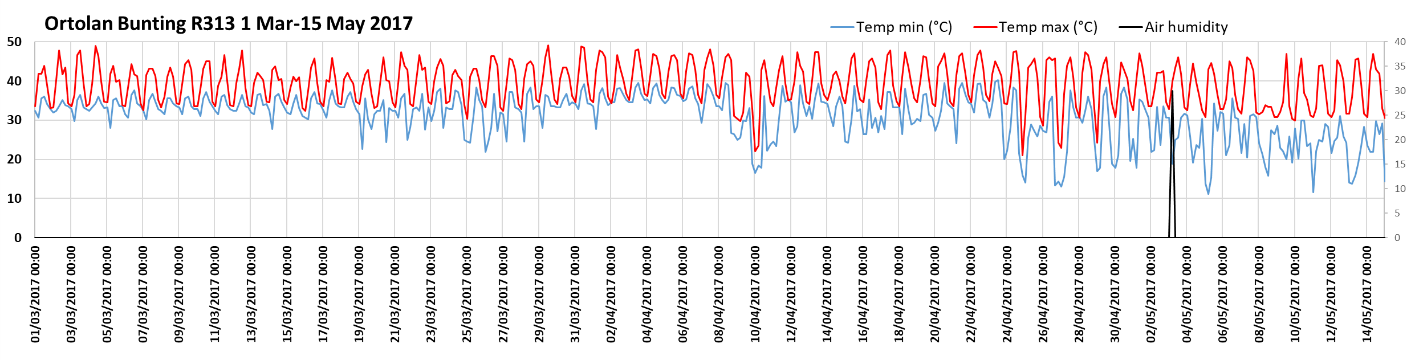


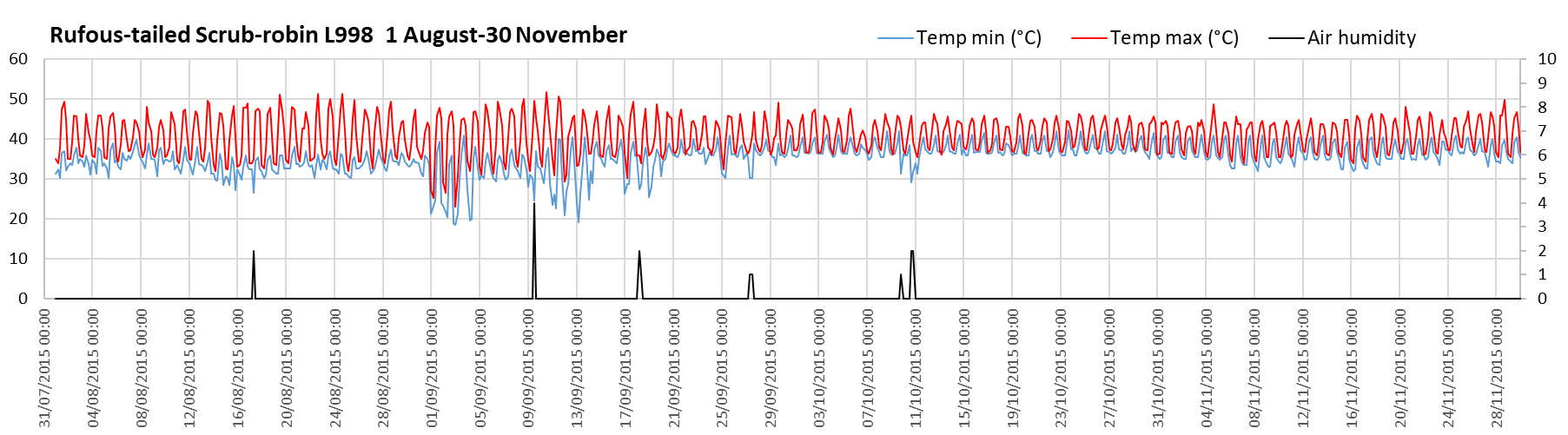


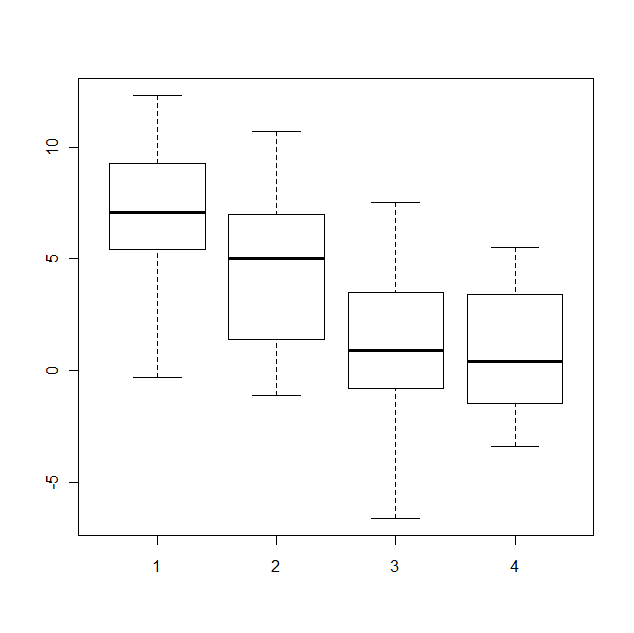
Figure S3. Boxplot reporting the differences (average, box of 25^th^ and 75^th^ quartiles, and 5%-95% confidence intervals) in early morning temperatures (within four hours after sunrise) recorded on a day of prolonged nocturnal flight compared to the temperature at the same hour the previous day or the next day without prolonged flight during daylight. Data for ortolan buntings in spring (left) and autumn (right).

Temperature differences (in °C)

Spring

(day before)

Autumn

(day after)

Autumn

(day before)

Spring

(day after)
